# Recombinant human lactoferrin enhances the efficacy of triple therapy in mice infected with *Helicobacter pylori*

**DOI:** 10.3892/ijmm.2015.2251

**Published:** 2015-06-17

**Authors:** YUPING YUAN, QINYI WU, GUOXIANG CHENG, XUEFANG LIU, SIGUO LIU, JUAN LUO, AIMIN ZHANG, LI BIAN, JIANQUAN CHEN, JIAJUN LV, XIANGQIAN DONG, GANG YANG, YUNZHEN ZHU, LANQING MA

**Affiliations:** 1 Yunnan Institute of Digestive Disease, Department of Digestive Diseases, The First Affiliated Hospital, Kunming Medical University, Kunming, Yunnan, P.R. China; 2 Laboratory for Conservation and Utilization of Bio-Resources, Yunnan University, Kunming, Yunnan, P.R. China; 3 Shanghai Jielong Bioengineering Co., Ltd., Shanghai, P.R. China; 4 Department of Pathology, The First Affiliated Hospital, Kunming Medical University, Kunming, Yunnan, P.R. China

**Keywords:** recombinant human lactoferrin, *Helicobacter pylori*, standard triple therapy regimen, bacterial eradication, inflammatory response

## Abstract

*Helicobacter pylori* (*H. pylori*) is a life-threatening pathogen which causes chronic gastritis, gastric ulcers and even stomach cancer. Treatment normally involves bacterial eradication; however, this type of treatment only has a rate of effectiveness of <80%. Thus, it is a matter of some urgency to develop new therapeutic strategies. Lactoferrin, a member of the transferrin family of iron-binding proteins, has been proven to be effective in removing a vast range of pathogens, including *H. pylori*. In the present study, we examined the effectiveness of recombinant human lactoferrin (rhLf) isolated from transgenic goats as a treatment for *H. pylori in vitro* and *in vivo*. For the *in vivo* experiments, BALB/c mice received an intragastric administration of 0.1 ml of a suspension of *H. pylori*. The mice were then divided into 4 groups: group A, treated with saline; group B, treated with 1.5 g of rhLF; group C, treated with the standard triple therapy regimen; and group D, treated with the standard triple therapy regimen plus.5 g of rhLF. Following sacrifice, the stomach tissues of the mice were histologically examined for the presence of bacteria. For the *in vitro* experiments, the bacteria were cultured in BHI broth and RT-qPCR and western blot analysis were carried out to determine the mRNA and protein levels of virulence factors (CagA and VacA) in the cultures. Our results revealed that rhLf not only inhibited the growth of *H. pylori*, but also suppressed the expression of two major virulence factors. Moreover, rhLf markedly increased bacterial eradication and effectively reduced the inflammatory response when combined with the standard triple therapy regimen. These results provide evidence supporting the use of rhLF as an adjuvant to traditional therapeutic strategies in the treatment of *H. pylori*.

## Introduction

*Helicobacter pylori* (*H. pylori*) is a spiral-shaped, Gram-negative bacterium that colonizes the human stomach. Patients infected by *H. pylori* usually develop asymptomatic chronic gastritis; there are no obvious clinical symptoms in the majority of *H. pylori* cases (1). *H. pylori* is considered a class I carcinogen that has been linked to gastric cancer by the World Health Organization (2). A recent study demonstrated that seroposi-tivity to *H. pylori* proteins is associated with an increased risk of biliary tract cancers (3). The current therapeutic regimen for eradicating *H. pylori* utilizes proton-pump inhibitors in combination with several antibiotics, such as clarithromycin, amoxicillin and metronidazole. Although this regimen has a promising rate of eradication, up to 35% of patients fail to respond to this treatment (4). Flurazolidone- and rifabutin-based therapies have also been used in the treatment of *H. pylori*. However, the limitations of and intolerance to these drugs make this therapeutic option less favorable (5). Other antibiotics have also been used in the treatment of *H. pylori*, including ciprofloxacin, fluoroquinolone and streptomycin (6,7). The rates of eradication in some studies have reached up to 80%; however, none have achieved a 90–95% eradication rate. Failures are associated particularly with discontinued treatment, primarily due to poor patient compliance and increased bacterial resistance (8).

Lactoferrin is a glycoprotein with multiple functions, and it is widely distributed in mucosal secretions, such as saliva, tears and seminal fluid (9). Lactoferrin has bacteriostatic, antiviral and antifungal properties (10,11). It has been reported that the bacteriostatic activity of lactoferrin is probably due to the sequestering of iron, which is essential for microorganism growth (12). Lactoferrin can also bind the lipopolysacchride of Gram-negative bacteria and disorganize and destabilize the bacterial surface, which in turn increases the ability of antibiotics to permeate the bacteria (13).

Clinical studies have demonstrated that lactoferrin increases the effectiveness of standard triple therapy for *H. pylori* (14–16). However, another study indicated that lactoferrin was ineffective in the treatment of *H. pylori* infection in humans (17). Currently, lactoferrin used in the treatment of *H. pylori* is mainly extracted from bovine milk, or recombinant human lactoferrin (rhLf) is extracted from bacteria, yeast or rice. In the present study, we examined the effectiveness of a novel rhLf derived from goat milk in the treatment of *H. pylori in vitro* and *in vivo*.

## Materials and methods

### Animal experiments

BABL/c mice (8–10 weeks of age) were obtained from the Animal Center, Kunming Medial University (Yunnan, China). The animals were housed at 21–22°C, with a 12-h light/dark cycle of 12:12 h. The protocol of the animal experiments was approved by the Animal Care and Use Committee of Kunming Medical University. All animal experiments complied with the Guide for the Care and Use of Laboratory Animals published by the US National Institute of Health (NIH Publication no. 8523, revised 1985).

The mice received an intragastric administration of 0.1 ml of a suspension of *H. pylori* ATCC 43504 (a gift from Professor Lu Hong, Shanghai Jiao Tong University School of Medicine, Shanghai, China) in brain-heart infusion broth (BHI broth; 10^8^ CFU/ml) once daily for 4 consecutive days. A total of 192 mice was divided into 4 groups as follows: mice in group A wre treated intragastrically with saline; mice in group B were treated intragastrically with 1.5 g of rhLf derived from the milk of transgenic goats (Shanghai Jielong Bioengineering Co., Ltd., Shanghai, China); mice in group C were treated intragastrically with the standard triple regimen [clarithromycin (Goldensun Pharmaceutical Co., Ltd., Xiamen, China), amoxicillin (Baker Norton Pharmaceutical Co., Ltd., Kunming, China) and a proton pump inhibitor (omeprazole; Harbin Pharmaceutical Co., Ltd., Harbin, China)]; and mice in group D were treated intragastrically with the triple therapy regimen plus 1.5 g of rhLf daily. Following 7 days of treatment, the mice were sacrificed by cervical dislocation, and a part of the stomach tissue of each mouse was immediately removed and quickly frozen in liquid nitrogen. The remaining parts of the stomach tissue were fixed in 10% formalin, dehydrated in graded ethyl alcohol and embedded in paraffin.

### Histological examination

The paraffin-embedded sections were examined for the presence of *H. pylori* by silver staining. The bacteria were characterized as a spiral rod of 3.0–0.5 mm, located adjacent to the gastric epithelium. The sections were also stained with hematoxylin and eosin (H&E), and were scored for their degrees of inflammation based on the intensity of neutrophilic and/or lymphocytic infiltration.

### Bacterial culture

*H. pylori* ATCC 43504 was inoculated onto a Columbia Agar base (CAB; Qingdao Hope Bio-Technology Co., Ltd., Qingdao, China) supplemented with 10% (v/v) fetal bovine serum (Thermo Fisher Scientific, Waltham, MA, USA), 1% (v/v) mixed antibiotics [trimethoprim, 5 g/l; polymyxin B sulfate, 3.85 g/l; amphotericin B, 10 g/l; and vancomycin, 10 g/l (all obtained from Solarbio Co., Beijing, China)] in a jar with an AnaeroPack Campylo sachet (Mitsubishi Gas Chemical Co., Tokyo, Japan) at 37°C for 3–5 days. For liquid culture, the plate-grown bacteria were inoculated into liquid BHI broth (Qingdao Hope Bio-Technology) supplemented with 10% (v/v) fetal bovine serum and 1% (v/v) mixed antibiotics, with an initial optical density at 600 nm (OD600) of 0.1.

### Effect of rhLf on the growth of H. pylori

After the *H. pylori* bacteria were allowed to grow for 18–20 h in the BHI liquid medium, the bacterial cultures were diluted in 10 ml of BHI liquid medium with rhLf (0–0.5 mg/ml) or desferrioxamine (DFO; Chengdu Best Reagent Co., Ltd., Chengdu, China) (0–50 *µ*M), to an initial OD600 of 0.1. The *H. pylori* broth cultures were then incubated at 37°C. Every 8 h, over a 64-h time period, a 200-*µ*l sample of each bacterial culture was isolated and the OD600 measured. The effect of ferric ammonium citrate (FAC; Chengdu Best Reagent Co., Ltd.) (0–50 *µ*M) in the presence of 0.5 mg/ml rhLf on bacterial growth was examined following the same procedure.

### Reverse transcription-quantitatve PCR (RT-qPCR)

Total RNA from the *H. pylori* cultures was isolated using TRIzol reagent (Takara, Dalian, China). cDNA was generated by the reverse transcription of total RNA using a PrimeScript™ RT reagent kit with a gDNA Eraser (Takara). Quantitative PCR (qPCR) was conducted using SYBR^®^Premix Ex Taq™ II (Takara) on a LightCycler^®^ 480 system (Roche Applied Science, Penzberg, Germany). 16S rRNA was used as an internal control. The primers used for PCR were as follows: cytotoxin-associated gene A (*CagA*) forward, 5′-GGGCGTG TTTGATGAGTCCT-3′ and reverse, 5′-TGT ATG TCG GTG GTG GTA GTG-3′; vacuolating cytotoxin A (*VacA*) forward, 5′-CCA ACT TAC CCA CAA ACA CC-3′ and reverse, 5′-TAG CCA ATT CAA ACA CGC TC-3′; urease (*Ure*) forward, 5′-TTC TTC TGC CTG GAG TGA TAG T-3′ and reverse, 5′-TTC TTC TGC CTG GAG TGA TAG T-3′; and 16S rRNA forward, 5′-TGT GGG AGA GGT AGG TGG AA-3′ and reverse, 5′-CAT CGT TTA GGG CGT GGA CT-3′.

### Western blot analysis

The bacteria were collected and washed with PBS. Total proteins were extracted using a radioimmu-noprecipitation assay (RIPA) lysis buffer (Beyotime Institute of Biotechnology, Beijing, China). The lysates (25 *µ*g) of total protein were loaded and run on a 10% polyacrylamide gel and transferred onto a polyvinylidene fluoride (PVDF) membrane.

The primary antibodies used were anti-CagA (sc-25766, rabbit polyclonal antibody; 1:3,000 dilution; Santa Cruz Biotechnology, Santa Cruz, CA, USA), anti-VacA (sc-25790, rabbit polyclonal antibody; 1:3,000 dilution; Santa Cruz Biotechnology), anti-Ure (sc-21016, rabbit polyclonal antibody; 1:3,000 dilution; Santa Cruz Biotechnology), and anti-GAPDH antibodies (GA1R, mouse monoclonal antibody; 1:5,000 dilution; Thermo Fisher Scientific, Waltham, MA, USA). The secondary antibody was a horseradish peroxidase-coupled anti-rabbit or mouse IgG (1:5,000 dilution; Santa Cruz Biotechnology). The membrane was exposed to Kodak X-Omat film (Kodak, Xiamen, China), and the film was then developed.

### Assays for tumor necrosis factor α (TNF-α) and interleukin-8 (IL-8)

After the stomach tissues were homogenized in liquid nitrogen, the homogenate was lysed on ice for 60 min in lysis buffer (BioTeke, Beijing, China). The protein concentration was determined in the supernatant by a Bradford protein assay kit (BioTeke) using bovine serum albumin as the standard. The levels of TNF-α and IL-8 in the supernatant were measured using commercially available ELISA kits (Xinbosheng Biotechnology Co., Ltd., Shenzhen, China). The values of TNF-α and IL-8 in the stomach tissues were normalized to the protein concentration.

### Statistical analysis

The statistical significance of the differences in gene and protein expression and in the levels of inflammatory factors were assessed by one-way analysis of variance (ANOVA), followed by a Student-Newman-Keuls test. The differences in bacterial eradication and inflammation were analyzed using the Wilcoxon rank-sum test. Data were analyzed using SPSS 11.0 software (SPSS, Inc., Chicago, IL, USA).

## Results

### rhLf inhibits the growth of H. pylori in vitro

**Figure 1:**
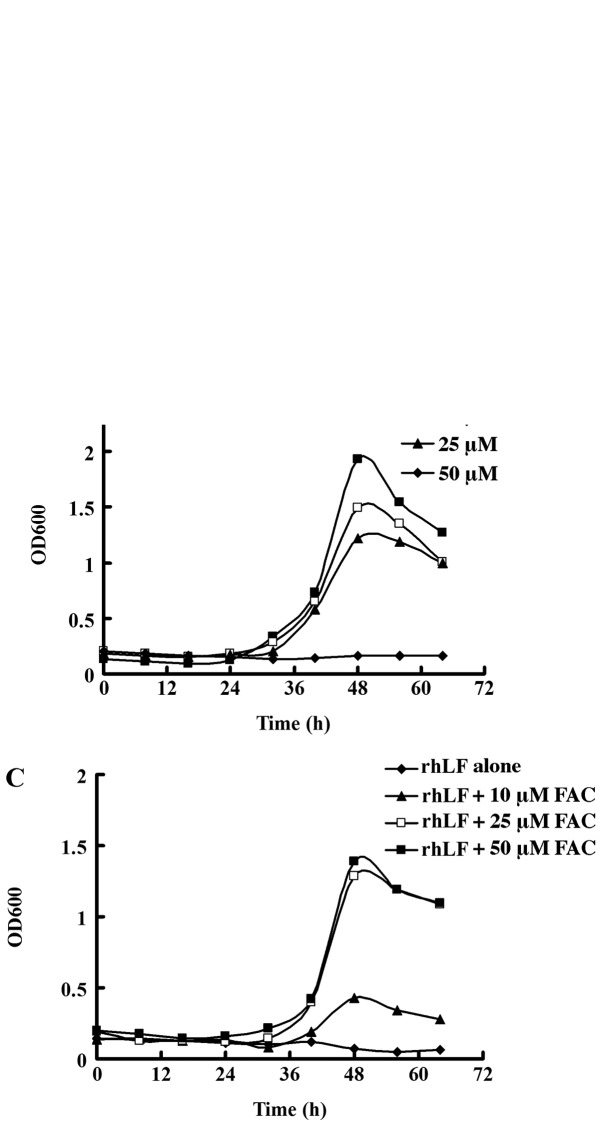
Recombinant human lactoferrin (rhLf) inhibits the growth of *H. pylori* in a concentration-dependent manner. (A and B) The growth of *H. pylori* was inhibited by (A) rhLf or (B) desferrioxamine (DFO) at different concentrations. (C) The inhibitory effects of rhLf (0.5 mg/ml) on the growth of *H. pylori* were repressed by the addition of ferric ammonium citrate (FAC).

It has been shown that rhLf obtained from bacteria, yeast, or rice is an effective adjuvant for use in the treatment of *H. pylori* (18,22). In order to determine the efficacy of rhLf derived from goat milk in the treatment of *H. pylori*, we first examined the effect of rhLf on the growth of *H. pylori*. As shown in Fig. 1A, treatment with rhLf suppressed the growth of *H. pylori* in a concentration-dependent manner. The growth of *H. pylori* was almost completely inhibited by rhLf at a concentration of 0.5 mg/ml. By contrast, *H. pylori* in the control group, which was not treated with rhLf, reached a growth peak after 48 h.

Lactoferrin has been shown to exhibit bacteriostatic activities, probably due to its ability to chelate iron (10). We found that similar to rhLf, DFO, a well-known chelating agent, also effectively suppressed the growth of *H. pylori* in a dose-dependent manner (Fig. 1B). By contrast, the addition of FAC significantly restored the growth of *H. pylori* (Fig. 1C). However, the growth of *H. pylori* was not restored to the same level as the control group even with a saturated iron supplement, suggesting that other mechanisms, aside from the chelation of iron, also participate in the bacteriostatic activity of rhLF.

### rhLf inhibits the expression of certain virulence factors in H. pylori

**Figure 2:**
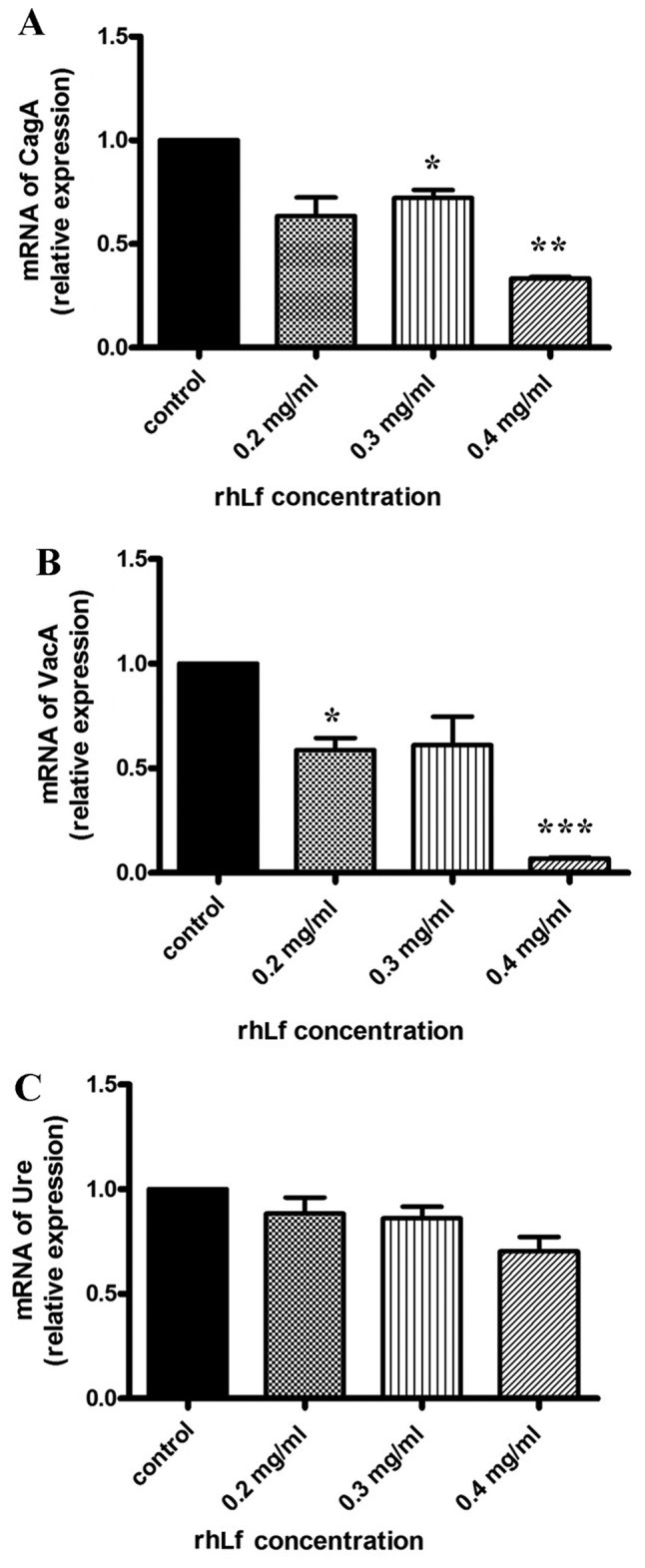
Recombinant human lactoferrin (rhLf) inhibits the expression of virulence factors of *H. pylori in vitro*. The mRNA levels of these virulence factors were determined by RT-qPCR. (A) Cytotoxin-associated gene A (*CagA*), (B) vacuolating cytotoxin A (*VacA*), and (C) urease (*Ure*). ^*^P<0.05, ^**^P<0.01 and ^***^P<0.001.

**Figure 3:**
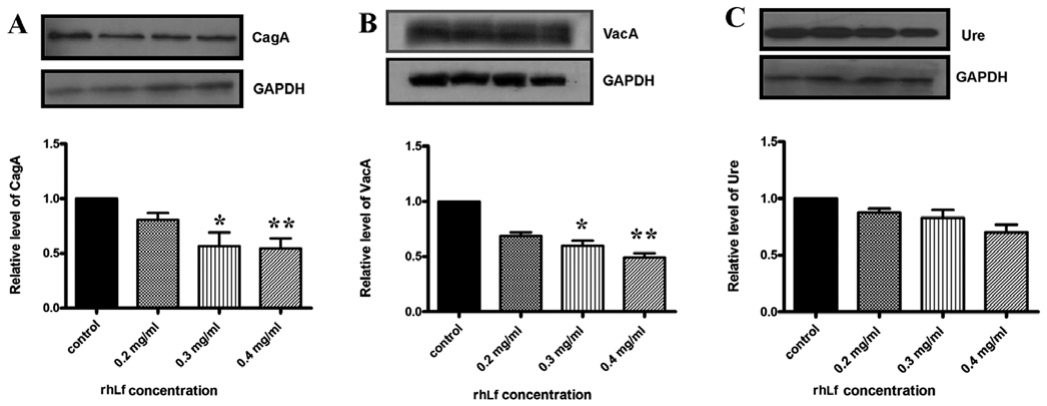
Recombinant human lactoferrin (rhLf) inhibits the protein levels of virulence factors of *H. pylori in vitro*. The protein levels of these virulence factors were determined by western blot analysis. The blots shown here are representative of 3 independent experiments. The lower panels show the quantification of the protein levels. (A) Cytotoxin-associated gene A (CagA), (B) vacuolating cytotoxin A (VacA) and (C) urease (Ure). ^*^P<0.05 and ^**^P<0.01.

We then determined whether rhLf affects the expression of 3 major virulence factors (*CagA*, *VacA* and *Ure*) by RT-qPCR. As shown in Fig. 2, a significant decrease in the mRNA levels of 2 of these virulence factors (*CagA* and *VacA*) following treatment with 0.4 mg/ml of rhLf was noted. The results were further confirmed by western blot analysis (Fig. 3). The protein levels of CagA and VacA were suppressed following treatment with 0.4 mg/ml of rhLf (Fig. 3A and B). By contrast, rhLf did not affect the expression of Ure (Fig. 3C). These results suggest that lactoferrin suppresses the expression of 2 major pathogenic virulence factors of *H. pylori*.

### Effects of rhLf on the eradication of H. pylori in vivo

**Figure 4:**
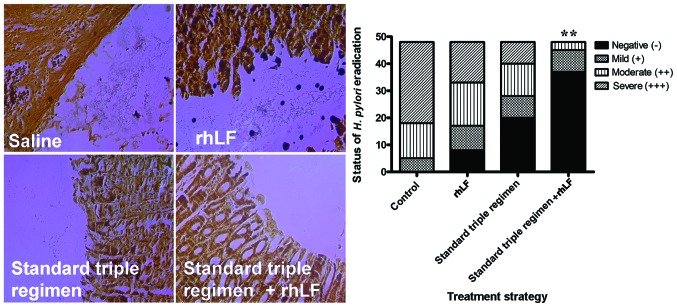
Effects of recombinant human lactoferrin (rhLf) on the eradication of *H. pylori in vivo*. The mice were inoculated with *H. pylori* for 4 days, and then received treatment for 7 days; subsequently, stomach tissue was collected. The presence of *H. pylori* was detected by silver staining (left panel). The distribution of *H. pylori* in the stomach tissue is depicted (right panel) (n=48, in each group). ^**^P<0.01 vs. the group treated with standard triple therapy regimen.

In the present study, the infected mice were divided into 4 groups: those treated with saline, rhLf, the standard triple therapy regimen, or the standard triple therapy regimen plus rhLf (quadruple treatment). To determine the efficiency of rhLf in the eradication of *H. pylori in vivo*, we performed semi-qualitative analysis of the distribution of *H. pylori* in the stomach tissues; silver staining was applied to the formalin-embedded tissue sections. As shown in Fig. 4, the number of *H. pylori* bacteria was significantly higher in the rhLf group than in the group which received the standard triple therapy regimen. However, quadruple treatment exerted a profound protective effect against the bacteria, as demonstrated by the complete absence of *H. pylori* bacteria in the silver-stained histological sections.

### Effects of rhLf on the inflammatory response in mice infected with H. pylori

**Figure 5:**
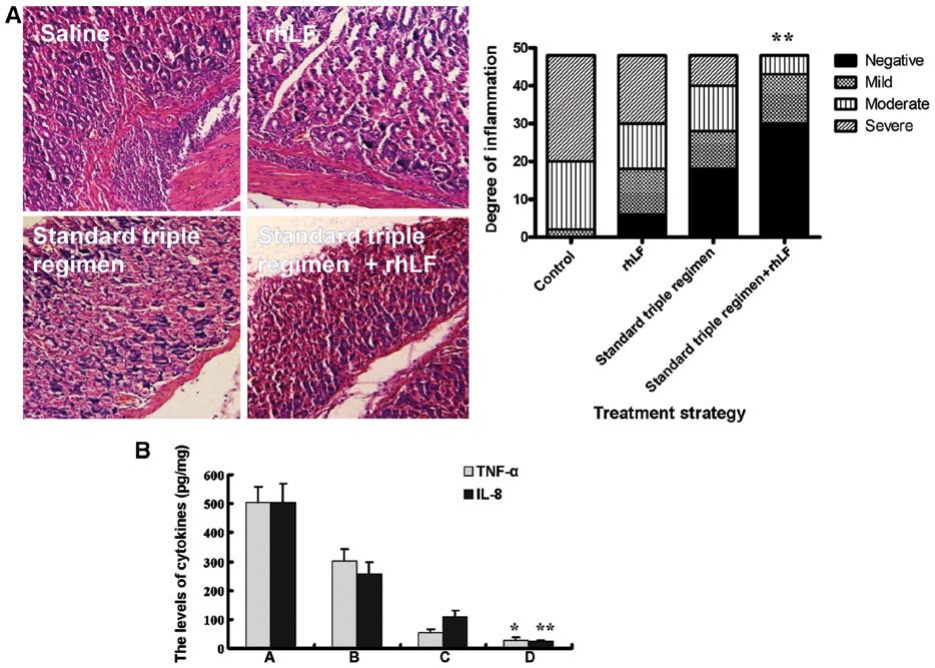
Effects of recombinant human lactoferrin (rhLf) on the inflammatory response in mice infected with *H. pylori*. (A) Stomach tissues from the mice infected with *H. pylori* were assessed by H&E staining (left panel). The inflammation classified by the histological score was measured (right panel) (n=48 in each group). (B) The levels of tumor necrosis factor α (TNFα) and interleukin 8 (IL-8) in the stomach tissues were determined by ELISA. Bars labeled A represent the control, bars labeled B represent the rhLF-treated group, bars labeled C represent the group treated with the standard triple therapy regimen, and bars labeled D represent the group treated with the standard triple therapy regimen + rhLF. ^*^P<0.05 and ^**^P<0.01 vs. the group treated with standard triple therapy regimen alone.

To determine the extent of inflammation, the stomach tissues from the mice in the 4 groups were harvested and stained with H&E. The group treated with rhLf alone exhibited a similar degree of gastric inflammation to the group treated with saline (control group), and the standard triple therapy regimen significantly reduced inflammation compared with these 2 groups. Notably, quadruple treatment reduced inflammation to a greater extent than the triple therapy regimen (Fig. 5A). Subsequently, we determined the levels of the inflammatory cytokines TNF-α and IL-8 in the stomach tissues (Fig. 5B). Unlike the standard triple therapy regimen, which markedly inhibited the increase in the TNF-α and IL-8 levels induced by the bacterium, treatment with rhLf alone resulted in only a slight decrease in the TNF-α and IL-8 levels (Fig. 5B). However, both the TNF-α and IL-8 levels markedly decreased in the group that received the quadruple treatment, compared to the group treated with the the triple therapy regimen alone. These results suggest that the addition of rhLf to the triple therapy regimen further inhibits the inflammatory response.

## Discussion

*H. pylori* is one of the most prevalent bacteria; it infects the gastric mucosa, and plays an etiological role in gastritis and peptic ulcer disease. *H. pylori* infection is usually asymptomatic and persistent, and thus has a high rate of prevalence (19). Previous studies have demonstrated that triple therapy with amoxicillin, clarithromycin and a proton pump inhibitor for 7 days led to a pooled eradication rate of <80% (16,20,21). We are currently facing two great challenges: the emerging multiple drug resistance of *H. pylori*, and the very limited number of alternative antibiotics. As a result, the development of novel treatment strategies is both necessary and urgent.

The bacteriostatic ability of lactoferrin, as an iron carrier, has been proven to be effective in the treatment of a variety of pathogens, including *H. pylori* (10). In the present study, we examined the effectiveness of rhLf in the treatment of *H. pylori in vitro* and *in vivo*. rhLf inhibited the growth of *H. pylori* in a concentration-dependent manner *in vitro*, which is consistent with the results of a previous study (22). Our results confirm that iron chelation seems to be one of the mechanisms underlying the inhibition of the growth of *H. pylori*. Our results demonstrated that rhLf decreased the expression of 2 major virulence factors, *CagA* and *VacA*. CagA is encoded by the *CagA* gene which is located in the *cag* pathogenicity island (23). Following the attachment of *H. pylori* to host gastric epithelial cells, CagA is injected into cells through the type IV secretion system (24). The presence and injection of CagA triggers host immune responses, including the secretion of IL-8 and the recruitment of various inflammatory factors (25). The other well-studied virulence factor of *H. pylori* is VacA, which is internalized into host epithelial cells through endocytosis (26). VacA causes massive vacuolation in epithelial cells and disrupts normal cell function, including endosome and lysosome activity, and cytoskeleton-dependent functions (27). However, whether the inhibitory effects of rhLf on the expression of *CagA* and *VacA* are associated with its iron sequestration remains unknown.

In this study, the analysis of the stomach tissues by silver staining revealed that treatment with rhLf alone failed to eradicate bacteria in the *H. pylori*-infected mice. However, quadruple treatment (triple therapy regimen plus rhLF) eradicated the bacteria more effectively than the triple therapy regimen. Indeed, it has been noted previously that treatment with lactoferrin over a 7-day period does not have an effect on the elimination of *H. pylori* (17), whereas the addition of lactoferrin improves the efficacy of standard triple therapy against *H. pylori* infection in human subjects (14–16). Likewise, rhLf alone did not attenuate the inflammatory response in *H. pylori*-infected mice. However, the inflammatory response in mice infected with *H. pylori* was markedly reduced by the standard triple therapy regimen plus rhLf, compared to the triple regimen alone. Taken together, our results demonstrate the efficacy of rhLf as an adjuvant in the treatment of *H. pylori* in mice.

## References

[1] Bytzer P, Dahlerup JF, Eriksen JR, Jarbøl DE, Rosenstock S, Wildt S, Danish Society for Gastroenterology (2011). Diagnosis and treatment of Helicobacter pylori infection. Dan Med Bull.

[2] Olokoba AB, Obateru OA, Bojuwoye MO (2013). Helicobacter pylori eradication therapy: A review of current trends. Niger Med J.

[3] Murphy G, Michel A, Taylor PR, Albanes D, Weinstein SJ, Virtamo J, Parisi D, Snyder K, Butt J, McGlynn KA (2014). Association of seropositivity to Helicobacter species and biliary tract cancer in the ATBC study. Hepatology.

[4] Leung WK, Graham DY (2002). Rescue therapy for Helicobacter pylori. Curr Treat Options Gastroenterol.

[5] Gisbert JP, Pajares JM (2002). Review article: Helicobacter pylori ‘rescue’ regimen when proton pump inhibitor-based triple therapies fail. Aliment Pharmacol Ther.

[6] Dresner D, Coyle W, Nemec R, Peterson R, Duntemann T, Lawson JM (1996). Efficacy of ciprofloxacin in the eradication of Helicobacter pylori. South Med J.

[7] Sanaka M, Kuyama Y, Yamanaka M, Iwasaki M (1999). Decrease in serum concentrations of Helicobacter pylori IgG antibodies during antituberculosis therapy: the possible eradication by rifampicin and streptomycin. Am J Gastroenterol.

[8] Kusters JG, van Vliet AH, Kuipers EJ (2006). Pathogenesis of Helicobacter pylori infection. Clin Microbiol Rev.

[9] Vorland LH (1999). Lactoferrin: a multifunctional glycoprotein. APMIS.

[10] Farnaud S, Evans RW (2003). Lactoferrin: a multifunctional protein with antimicrobial properties. Mol Immunol.

[11] Ikeda M, Nozaki A, Sugiyama K, Tanaka T, Naganuma A, Tanaka K, Sekihara H, Shimotohno K, Saito M, Kato N (2000). Characterization of antiviral activity of lactoferrin against hepatitis C virus infection in human cultured cells. Virus Res.

[12] Ellison RT, Giehl TJ, LaForce FM (1988). Damage of the outer membrane of enteric Gram-negative bacteria by lactoferrin and transferrin. Infect Immun.

[13] Drago-Serrano ME, de la Garza-Amaya M, Luna JS, Campos-Rodriguez R (2012). Lactoferrin-lipopolysaccharide (LPS) binding as key to antibacterial and antiendotoxic effects. Int Immunopharmacol.

[14] Di Mario F, Aragona G, Bò ND, Ingegnoli A, Cavestro GM, Moussa AM, Iori V, Leandro G, Pilotto A, Franzè A (2003). Use of lactoferrin for Helicobacter pylori eradication. Preliminary results. J Clin Gastroenterol.

[15] Sachdeva A, Nagpal J (2009). Meta-analysis: efficacy of bovine lacto-ferrin in Helicobacter pylori eradication. Aliment Pharmacol Ther.

[16] de Bortoli N, Leonardi G, Ciancia E, Merlo A, Bellini M, Costa F, Mumolo MG, Ricchiuti A, Cristiani F, Santi S (2007). Helicobacter pylori eradication: a randomized prospective study of triple therapy versus triple therapy plus lactoferrin and probiotics. Am J Gastroenterol.

[17] Guttner Y, Windsor HM, Viiala CH, Marshall BJ (2003). Human recombinant lactoferrin is ineffective in the treatment of human Helicobacter pylori infection. Aliment Pharmacol Ther.

[18] Miehlke S, Reddy R, Osato MS, Ward PP, Conneely OM, Graham DY (1996). Direct activity of recombinant human lactoferrin against Helicobacter pylori. J Clin Microbiol.

[19] Peek RM, Blaser MJ (2002). Helicobacter pylori and gastrointestinal tract adenocarcinomas. Nat Rev Cancer.

[20] Vergara M, Vallve M, Gisbert JP, Calvet X (2003). Meta-analysis: comparative efficacy of different proton-pump inhibitors in triple therapy for Helicobacter pylori eradication. Aliment Pharmacol Ther.

[21] Altintas E, Sezgin O, Ulu O, Aydin O, Camdeviren H (2004). Maastricht II treatment scheme and efficacy of different proton pump inhibitors in eradicating Helicobacter pylori. World J Gastroenterol.

[22] Dial EJ, Hall LR, Serna H, Romero JJ, Fox JG, Lichtenberger LM (1998). Antibiotic properties of bovine lactoferrin on Helicobacter pylori. Dig Dis Sci.

[23] Censini S, Lange C, Xiang Z, Crabtree JE, Ghiara P, Borodovsky M, Rappuoli R, Covacci A (1996). cag, a pathogenicity island of Helicobacter pylori, encodes type I-specific and disease-associated virulence factors. Proc Natl Acad Sci USA.

[24] Christie PJ, Vogel JP (2000). Bacterial type IV secretion: conjugation systems adapted to deliver effector molecules to host cells. Trends Microbiol.

[25] Fischer W, Puls J, Buhrdorf R, Gebert B, Odenbreit S, Haas R (2001). Systematic mutagenesis of the Helicobacter pylori cag pathogenicity island: essential genes for CagA translocation in host cells and induction of interleukin-8. Mol Microbiol.

[26] Papini E, Satin B, Norais N, de Bernard M, Telford JL, Rappuoli R, Montecucco C (1998). Selective increase of the permeability of polarized epithelial cell monolayers by Helicobacter pylori vacuolating toxin. J Clin Invest.

[27] Cover TL, Blanke SR (2005). Helicobacter pylori VacA, a paradigm for toxin multifunctionality. Nat Rev Microbiol.

